# Towards a shared vision for measureable and meaningful health outcomes for children and young people with neurodisability: qualitative research, Delphi survey, systematic review, and stakeholder prioritisation

**DOI:** 10.1186/1745-6215-16-S1-O4

**Published:** 2015-05-29

**Authors:** Christopher Morris, Astrid Janssens, Amanda Allard, Joanne Thompson Coon, Valerie Shilling, Richard Tomlinson, Jane Williams, Andrew Fellowes, Morwenna Rogers, Karen Allen, Bryony Beresford, Colin Green, Crispin Jenkinson, Alan Tennant, Stuart Logan

**Affiliations:** 1 University of Exeter Medical School, University of Exeter, Exeter, UK; 2 Council for Disabled Children, National Children’s Bureau, London, UK; 3 Royal Devon and Exeter NHS Foundation Trust, Exeter, UK; 4 Nottingham University Hospitals NHS Trust, Nottingham, UK; 5 Social Policy Research Unit, University of York, York, UK; 6 Nuffield Department of Population Health, University of Oxford, Oxford, UK; 7 Department of Rehabilitation Medicine, University of Leeds, Leeds, UK

## Objective

To seek a shared vision between families and clinicians regarding key aspects of health as outcomes, beyond mortality and morbidity, for children and young people with neurodisability. To appraise the appropriateness and measurement properties of multidimensional patient reported outcome measures (PROMs) to assess the outcome domains.

## Methods

Relevant outcomes were identified from (i) qualitative research with children and young people with neurodisability and parent carers, (ii) Delphi survey with health professionals, and (iii) systematic review of PROMs. The International Classification of Functioning Disability and Health provided a common language to code aspects of health. A stakeholder group participated in a prioritisation Q-sort task. Participants 54 children and young people with neurodisability and 53 parent carers participated in either focus groups or interviews; 262 multidisciplinary health professionals took part in one or more rounds of a Delphi survey. 15 stakeholders participated in a consensus meeting: 3 young people, 5 parent carers, and 7 multidisciplinary health professionals.

## Results

The qualitative study and Delphi survey suggested a range of aspects of health that are important to service users and targeted by health professionals. There was partial but not complete overlap. Key outcome areas prioritised were: communication, emotional wellbeing, pain, sleep, mobility, self-care, independence, mental health, social activities; behaviour, toileting, and safety were also important to many parents. No single multidimensional PROM was identified that captured all the key aspects of health. Evidence was lacking of one or more measurement properties for all candidate PROMs in children and young people with neurodisability, and especially for preference-based measures.

## Conclusions

This research proposes a core suite of outcome domains for children and young people with neurodisability that can be used to assess health services routinely and in trials. Further work is required to produce a single PROM to measure these outcomes efficiently across neurodisability.

